# Investigation of immune-related diseases using patient-derived induced pluripotent stem cells

**DOI:** 10.1186/s41232-023-00303-4

**Published:** 2023-10-24

**Authors:** Hirofumi Shoda, Bunki Natsumoto, Keishi Fujio

**Affiliations:** 1 Department of Allergy and Rheumatology, Graduate School of Medicine, The University of Tokyo, 7-3-1 Hongo, Bunkyo-Ku, Tokyo, 113-8655 Japan; 2 Laboratory for Autoimmune Diseases, RIKEN Center for Integrative Medical Sciences, 1-7-22 Suehirocho, Tsurumi-Ku, Yokohama, Kanagawa 230-0045 Japan

**Keywords:** Induced pluripotent stem cell, Interferonopathy, Autoimmune disease, Autoinflammatory disease, Systemic lupus erythematosus

## Abstract

The precise pathogenesis of immune-related diseases remains unclear, and new effective therapeutic choices are required for the induction of remission or cure in these diseases. Basic research utilizing immune-related disease patient-derived induced pluripotent stem (iPS) cells is expected to be a promising platform for elucidating the pathogenesis of the diseases and for drug discovery. Since autoinflammatory diseases are usually monogenic, genetic mutations affect the cell function and patient-derived iPS cells tend to exhibit disease-specific phenotypes. In particular, iPS cell-derived monocytic cells and macrophages can be used for functional experiments, such as inflammatory cytokine production, and are often employed in research on patients with autoinflammatory diseases.

On the other hand, the utilization of disease-specific iPS cells is less successful for research on autoimmune diseases. One reason for this is that autoimmune diseases are usually polygenic, which makes it challenging to determine which factors cause the phenotypes of patient-derived iPS cells are caused by. Another reason is that protocols for differentiating some lymphocytes associated with autoimmunity, such as CD4^+^T cells or B cells, from iPS cells have not been well established. Nevertheless, several groups have reported studies utilizing autoimmune disease patient-derived iPS cells, including patients with rheumatoid arthritis, systemic lupus erythematosus (SLE), and systemic sclerosis. Particularly, non-hematopoietic cells, such as fibroblasts and cardiomyocytes, differentiated from autoimmune patient-derived iPS cells have shown promising results for further research into the pathogenesis. Recently, our groups established a method for differentiating dendritic cells that produce interferon-alpha, which can be applied as an SLE pathological model. In summary, patient-derived iPS cells can provide a promising platform for pathological research and new drug discovery in the field of immune-related diseases.

## Background

**Fig. 1:**
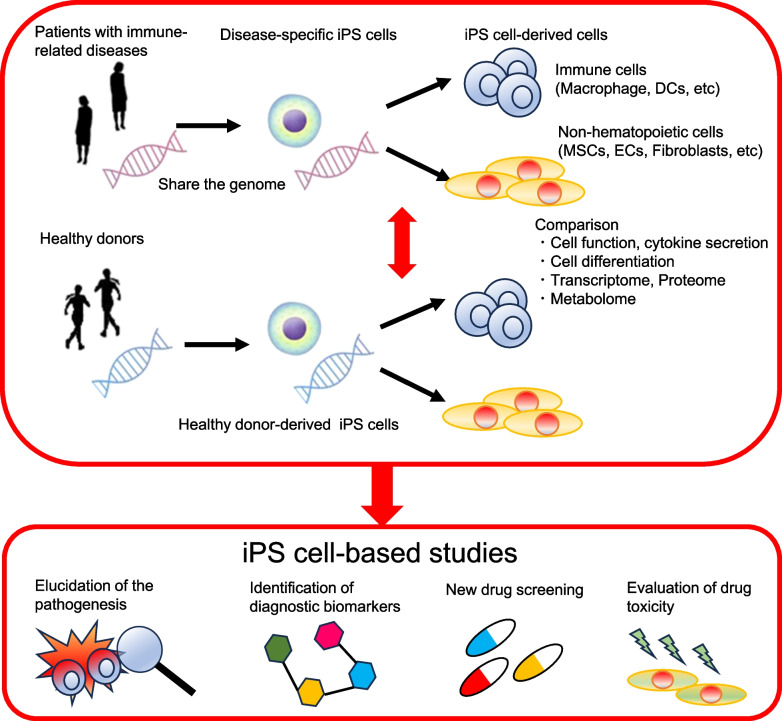
A schematic summary of the investigation of immune-related diseases using patient-derived iPS cells. Abbreviations are the same as the main document

Investigating human immune-related diseases using induced pluripotent stem (iPS) cells presents a novel and promising field of study. In the fields of hematological and neurological diseases, particularly monogenic ones, patient-derived iPS cells have helped to elucidate the pathogenesis of these diseases, and drug discovery studies are currently in progress [1–3]. We suggest an advantage of utilizing iPS cells for studying human immune-related diseases. In particular, patient-derived iPS cells retain the same genetic background as these patients, allowing for recurrent analysis of genetic effects on cellular functions. Therefore, studies of monogenic immune-related diseases are good candidates for employing patient-derived iPS cells [4, 5]. As immune cells typically undergo differentiation from precursor or naïve cells to mature cells, it is possible to observe the cell differentiation process by using iPS cell-derived immune cells. In human disease studies, ethical concerns often restrict the collection of patient cells and tissues. In particular, the analysis of inflamed tissue samples is essential to comprehend the pathogenesis; however, these tissue samples are often difficult to obtain. While animal studies provide valuable models for human diseases, there are differences in the mouse genome, immune system, and disease models compared to humans [6]. With iPS cell technology, researchers can readily acquire human cells and investigate these diseases using human-derived samples. Moreover, iPS cell-based studies have proposed new diagnostic biomarkers and contributed to the discovery of new therapeutic targets [7]. Drug screening based on patient-derived iPS cells has been conducted in the field of neurology [8]. Familial dysautonomia (FD) is caused by a point mutation in I kappa B (IkB) kinase complex-associated protein (IKBKAP)8. Large-scale drug screening identified compounds that improved neuronal differentiation and migration by using FD patient-derived iPS cells [9]. In this way, investigating human immune-related diseases using iPS cells offers distinct advantages over conventional research methods. In this review, we discuss the application of iPS cells in the study of human immune-related diseases, with an emphasis on autoimmune diseases (Fig. 1).

## Main text

### iPS cell-derived immune cells

To investigate the pathogenesis of autoimmune diseases, it is crucial to establish in vitro disease models using iPS cell-derived cells. Given the complexity of the immune system, numerous types of cells are implicated in immune-related diseases, and the interactions among these cells play critical roles in their pathogenesis. While the analysis of iPS cell-derived immune cells might provide partial insights, it can still yield vital clues into understanding immune-related diseases. First, we will review the types of immune cells which can be differentiated from iPS cells. The protocols for differentiating iPS cell-derived immune cells are somewhat analogous to those used for embryonic stem cells [10, 11]. Although iPS cells exhibit pluripotency, differentiating them into immune cells is intricate, and to date, not all immune cell types can be successfully differentiated from iPS cells. For instance, the differentiation of T cells requires an in vivo thymic microenvironment, and CD4^+^ T cells have not yet been differentiated from iPS cells [12]. In contrast, immune cells associated with innate immunity can be differentiated from iPS cells. Notably, there is growing interest in iPS cell-derived natural killer and natural killer T cells for potential applications in cell therapy targeting neoplasms [12–14]. CD8^+^ T cells can be differentiated from iPS cells. Since mature T cells contain a rearranged T cell receptor (TCR) gene, iPS cells derived from peripheral T cells carry a specific TCR gene [12, 14, 15]. For immunotherapy, iPS cell-derived T cells equipped with an antigen-specific TCR or chimeric antigen receptor could offer promising for targeted cell destruction or depletion [14–16]. Yet, it is worth noting that these iPS cell-derived T and B cells have not been utilized extensively in studying immune-related diseases.

Monocytes and macrophages differentiated from iPS cells have frequently been employed to analyze the phenotypes of immune-related diseases. Various protocols exist for iPS cell-derived macrophage differentiation. Similar to the differentiation protocol for monocyte-derived macrophages (MDMs) from peripheral blood monocytes, macrophages (MPs) can be differentiated from iPS-derived myeloid lineage cells with M-CSF [17, 18]. Another group has reported the development of interferon (IFN)-alpha-secreting myeloid cells derived from iPS cells [19]. MDMs and MPs derived from iPS cells were thought to exhibit comparable phenotypes, functions, and transcriptomes. It is suggested that iPS cell-derived MPs resemble embryonic-origin macrophages, whereas peripheral blood-derived MDMs align more closely with those through a definitive hematopoiesis process [4]. Moreover, these MPs can undergo further differentiation into tissue-resident macrophages, serving as a model for a local inflammation [4]. Since these macrophages can produce pro-inflammatory cytokines, the major drivers of many immune-mediated diseases, iPS cell-derived macrophage-lineage cells have been utilized in researching monogenic autoinflammatory diseases. Dendritic cells (DCs) can also be differentiated from iPS cell-derived monocytes with granulocyte–macrophage colony-stimulating factor (GM-CSF) and interleukin (IL)-4 [14, 17]. These iPS cell-derived DCs retain primary DC functions, such as antigen presentation, cytokine production, and T cell stimulation, and are thought to be similar to myeloid DCs. Recently, our group has successfully developed iPS cell-derived CD123^+^ DCs [20]. These CD123^+^DCs can secret significant amounts of IFN-alpha in response to nucleic acid stimulation, which represents the function of plasmacytoid DCs. These iPS cell-derived CD123^+^DCs hold promise for investigating virus-induced responses or interferonopathies.

### iPS cell-based studies of autoinflammatory diseases

Immune-related diseases arise from the dysregulation of the immune system. Among them, autoinflammatory diseases are characterized by the pathogenesis in which pro-inflammatory cytokines play pivotal roles, and the genetic mutations related to immune-associated genes directly cause diseases [21]. For instance, periodic fever is caused by the excess secretion of pro-inflammatory cytokines, and genetic mutation in related genes leads to the dysregulation of inflammasome [21]. As autoinflammatory diseases are usually monogenic, patient-derived iPS cells can exhibit disease-related phenotypes [4, 5]. Moreover, autoinflammatory disease patient-derived iPS cells can be employed for both biomarker discovery and drug screening. By modifying the mutated genes in iPS cells, the roles of these mutations can be clarified through iPS cell-based analysis. Since the research on patient-derived iPS cells in periodic fever syndrome (excluding interferonopathies) was comprehensively reviewed elsewhere [4, 5], this review focuses on the major studies and findings related to other autoinflammatory diseases.

### Periodic fever syndrome

Monocytes and macrophages are major producers of pro-inflammatory cytokines and can be differentiated from periodic fever patient-derived iPS cells to analyze their phenotypic attributes. Macrophages derived from iPS cells of familial Mediterranean fever (FMF) patients with the *MEFV* gene mutations displayed pro-inflammatory phenotypes. These cells produced elevated levels of IL-1, IL-18, and chemokine (C–C motif) ligands (CCL)4 and amplified the formation of the inflammasome [18]. Chronic infantile neurological cutaneous and articular (CINCA) syndrome is another form of periodic fever, caused by gene mutations in *NLRP3* and *NLRP4*. Macrophages differentiated from CINCA patient-derived iPS cells with the *NLRP3* mutation exhibited abnormal IL-1 secretion [22]. Similarly, monocytes differentiated from CINCA syndrome patient-derived iPS cells with the *NLRP4* mutation demonstrated excessive secretion of IL-1 and IL-18, and *NLRC4* knockout in iPS cells led to notable reductions in pro-inflammatory cytokine secretion [23]. Blau syndrome is a juvenile-onset granulomatous disease, also referred to as early-onset sarcoidosis, caused by *NOD2* gene mutation. Macrophages differentiated from Blau syndrome patient-derived iPS cells presented increased Nuclear factor Kappa B (NFkB) activity and heightened pro-inflammatory cytokine secretion in response to IFN-gamma [24]. Remarkably, when the *NOD2* mutation was corrected via genome editing in iPS cells, there was a decrease in NFkB activity and a reduced pro-inflammatory cytokine secretion in response to IFN-gamma [24]. In this way, monocytes and macrophages differentiated from autoinflammatory disease patient-derived iPS cells exhibited pronounced pro-inflammatory phenotypes, making them accurate models for the diseases.

### Newly identified monogenic autoinflammatory diseases

Futhermore, patient-derived iPS cell-based studies have been instrumental in functionally analyzing newly identified autoinflammatory diseases. For instance, a patient with a missense mutation of *NFKBIA*, which results in the L34P IkB-alpha variant, concurrently exhibited symptoms of both autoinflammatory disease and immunodeficiency. Macrophages differentiated from this patient-derived iPS cells displayed increased IL-1 secretion, attributable to reduced NFkB inhibition [25]. Additionally, the Oligoadenylate synthetase (OAS)1 gain-of-function variant was identified as the causal mutation of OAS1-associated polymorphic autoinflammatory immunodeficiency (OPAID). Macrophages differentiated from the iPS cells with OAS1 mutations demonstrated diminished cell adhesion and phagocytosis [26]. In this way, patient-derived iPS cells have proven invaluable in advancing research on newly identified autoinflammatory diseases.

### Interferonopathy

Interferonopathies are monogenic diseases resulting from overexpression of type I IFNs and are considered autoinflammatory diseases [27, 28]. Mutations in genes related to nucleic acid processing, recognition, and IFN-related signaling have been identified as the causes of these diseases [27]. Clinically, interferonopathy is characterized by both neurological and inflammatory phenotypes. For example, Aicardi-Goutieres syndrome (AGS), STING-associated infantile vasculitis (SAVI), Interferon stimulated gene (ISG)15 deficiency, and pseudo-Toxoplasmosis, Rubella, Cytomegalovirus, and Herpes simplex virus (TORCH) syndrome, are known as typical interferonopathies [28]. Nakajo-Nishimura syndrome (NNS)/proteasome-associated autoinflammatory syndrome is also classified as a type of interferonopathy. We have summarized published studies on interferonopathy-derived iPS cells in Table 1.

**Table 1:** List of the studies of interferonopathy patient-derived iPS cells

Author (Ref)	Disease	Gene	iPS cell-derived cell types	Key findings
Genova et al. [29]	AGS	*TREX1, RANSEH2B, IFIH1*	iPS cells	Differences in drug cytotoxicity of drugs due to genetic mutations
Ferraroet al. [30]	AGS	*TREX1*	iPS cells	iPS cell establishment
Ferraro et al. [31]	AGS	*RNASEH2B*	iPS cells	iPS cell establishment
Masneri et al. [32]	AGS	*IFIH1*	iPS cells	iPS cell establishment
Fuchs et al. [33]	AGS	*SAMHD1*	iPS cells	iPS cell establishment
Hanchen et al. [34]	AGS	*SAMHD1*	iPS cells	iPS cell establishment
Hanchen et al. [35]	AGS	*TREX1*	iPS cells	iPS cell establishment
Barnabei et al. [36]	SAVI	*STING*	iPS cells	iPS cell establishment
Mehta et al. [37]	SAVI	*STING*	iPS cells	iPS cell establishment
Honda-Ozaki et al. [38]	NNS	*PMSB8*	Myeloid cells	Pro-inflammatory cytokine overexpression via reactive oxygen species and p38 MAPK
Kase et al. [39]	NNS	*PMSB8*	Myeloid cells	Drug screening for inhibiting pro-inflammatory chemokine secretion

References and abbreviations are the same as the main document

AGS was first described by Aocardi and Goutieres in 1984 as a progressive familial encephalopathy accompanied by basal ganglia calcification and chronic cerebrospinal fluid pleocytosis. It manifests with central nervous system symptoms, including severe complications, such as psychomotor developmental delay, microcephaly, and epilepsy, appearing from infancy [40]. Several causal mutations have been reported, and these are linked with nucleic acid metabolism and its sensing system. Furthermore, AGS patients occasionally develop autoimmune disorders as a characteristic feature. Approximately, 30% of patients exhibit chilblains-like eruptions, and some cases have also shown the presence of anti-nuclear antibodies, anti-double stranded (ds) DNA antibodies, and manifestations like systemic lupus erythematosus (SLE) [40]. Various research groups have reported the establishment of AGS patient-derived iPS cells with *TREX1*, *RNASEH2B*, *SAMHD1*, and *IFIH1* gene mutations [29–35]. Genova et al. established three AGS patient-derived iPS cells and examined the cytotoxicity of several drugs [29]. Their findings revealed that iPS cells with an *IFIH1* mutation exhibit altered susceptibility to mepacrine. Moreover, the responses to thioguanine were varied; iPS cells with an *IFIH1* mutation were more sensitive, while those with an *RNASEH2B* mutation showed reduced sensitivity to thioguanine [29]. Therefore, the authors proposed that AGS patient-derived iPS cells could serve as a platform for exploring the efficacy of potential drugs for AGS. Our group has also recently generated iPS cells carrying the *IFIH1* R778H variant with genome editing. The melanoma differentiation-associated protein (MDA)5, encoded by *IFIH1*, acts as a cytoplasmic dsRNA receptor. CD123^+^ DCs differentiated from iPS cells with the IFIH1 778H variant secreted an elevated amount of IFN-alpha in response to dsRNA stimulation, and this overproduction of IFN-alpha was regulated by 2′-5′-Oligoadenylate Synthetase Like (OASL) [20]. We propose that this system holds potential as a tool for drug discovery in the context of interferonopathy.

SAVI is an autoinflammatory disease caused by gain-of-function mutations in the *TMEM173* gene. STING, which is encoded by *TMEM173*, resides in the endoplasmic reticulum membrane. It is dimerized and is activated by cGAMP, the second messenger of cyclic GMP-AMP synthase (cGAS), a dsDNA sensor located in the cytoplasm. This activation process subsequently triggers the interferon signature gene (ISG) via IRF3. Clinically, SAVI presents with symptoms like periodic fever, purpuric rashes and ulcers, and lymphocytic interstitial pneumonia [41]. From a pathological standpoint, inflammation of the vascular wall, neutrophil infiltration, and microthrombi are evident. In certain cases, the presence of anti-nuclear antibodies, anti-neutrophil cytoplasmic antibodies (ANCA), and anti-phospholipid antibodies can be detected. Moreover, the deposition of the immune complex on the vessel walls might also be observed. Although there have been two reports on the establishment of SAVI patient-derived iPS cells [36, 37], functional analysis of these cells has not been conducted to date.

Finally, we highlight pioneering research using NNS patient-derived iPS cells. NNS is an autoinflammatory disease caused by *Proteasome (Prosome macropain) subunit beta (PSMB)8* gene mutation [42, 43]. Dysfunction of the immunoproteasome triggers recurrent and progressive inflammatory reactions. In childhood, patients with NNS develop chilblain-like eruptions on their hands and feet, with the emergence of erythema nodosum-like eruption. Characteristic long knotty fingers, muscle atrophy, and emaciation gradually progress, and flexion contracture of the fingers and elbow joints may occur. Honda-Ozaki et al. successfully established NNS patient-derived iPS cells [38]. Monocytic cells differentiated from NNS patient-derived iPS cells exhibited elevated pro-inflammatory cytokine and chemokine secretion. Notably, these monocytic cells produced augmented levels of reactive oxygen species and displayed increased p38 MAPK activity. The authors demonstrated that an inhibitor of p38 MAPK and antioxidants effectively curtailed excessive cytokine and chemokine secretions. In addition, a high-throughput compound screening was conducted on monocytic cells differentiated from iPS cells with the *PSMB8* mutation. This led to the identification of a potent compound that inhibits cytokine and chemokine production [39]. In this way, iPS cell-based studies can elucidate the pathogenesis of diseases and serve as a foundation for the identification of novel therapeutic candidates for autoinflammatory diseases.

### iPS cell-based studies of autoimmune diseases

In contrast to autoinflammatory diseases, autoimmune diseases arise due to dysregulation of antigen-specific immune responses. Antigen-specific autoimmunity is roughly categorized into organ-specific and systemic responses [44]. For example, autoimmune thyroiditis represents an organ-specific autoimmune disease, characterized by the development of anti-thyroidal autoantibodies. In contrast, SLE typified a systemic autoimmune disease, distinguished by autoantibodies targeting ubiquitously expressed molecules, such as anti-nuclear and anti-dsDNA antibodies [44, 45]. In the pathogenesis of autoimmune diseases, both T and B cells have central roles, and loss of self-tolerance usually precedes the clinical onset of the diseases [44]. Although the precise causes of autoimmune diseases remain uncovered, both genetic and environmental factors contribute to their pathogenesis [44, 45]. In other words, autoimmune diseases are polygenic, with the accumulation of multiple low-impact causal variants facilitating autoimmune and pathogenic processes [46]. Autoinflammatory disease patient-derived iPS cells typically harbor a monogenic variant that potently influences inflammation [21]. Whereas in the case of autoimmune disease patient-derived iPS cells, individual risk gene effects tend to be nuanced, making disease phenotypes occasionally difficult to reproduce in iPS cell-based analyses, despite a genetic predisposition to autoimmunity. Given the polygenic nature of autoimmune diseases, identifying causal genes becomes a challenge, especially since disparities exist between healthy donor-derived and autoimmune disease patient-derived iPS cells. Environmental factors are acknowledged to significantly impact autoimmune disease pathogenesis. While the mechanisms by which environmental factors induce disease are intricate and challenging to replicate in vitro, their effects can be evaluated using iPS cell-based studies. For example, viral infections, which are prevalent environmental triggers in many autoimmune diseases, stimulate innate immune responses through their nucleic acids. By exposing iPS cell-derived immune cells to nucleic acids, differences in reactions to environmental factors between healthy controls and autoimmune disease patients can be assessed [20]. T and B cells are fundamental to the pathogenesis of autoimmune disease, with genetic risks manifesting cell-specific effects [47]. However, differentiation from iPS cells is limited mostly to CD8 + T cells, posing a significant challenge for establishing disease models in iPS cell-based research. Notably, many researchers have opted for differentiating iPS cells into mesenchymal cells, endothelial cells, and fibroblasts instead of immune cells, yielding promising outcomes. Despite the existing limitations in patient-derived iPS cell-based studies for autoimmune diseases, numerous compelling studies employing innovation strategies have been published, and we have summarized these works in Table 2.

**Table 2:** List of the studies of autoimmune disease patient-derived iPS cells

Author (Ref)	Disease	iPS cell-derived cell types	Key findings
Lee et al. [48]	RA	Teratoma, osteoblast	iPS cell establishment from FLS, Osteoblast differentiation
Rim et al. [49]	RA	iPS cells	iPS cell establishment from FLS
Wolnik et al. [50]	RA	iPS cells	iPS cell establishment from FLS
Lee et al. [51]	RA	Cardiomyocytes	Disfunction of RA-iPS cell derived cardiomyocyte
Kim et al. [52]	RA	iPS cells	Different metabolic profiles between RA and OA-iPS cells. Proliferation of RA-iPS cells by NAM
Kim et al. [53]	RA	Hepatocytes	Recapitulation of methotrexate hepatotoxicity
Layh-Schmitt et al. [54]	AS	Mesenchymal stem cells, osteoblasts, chondrocytes, adipocytes	Elevated expression of AS susceptibility genes associated with bone formation in AS-iPS cell-derived MSCs
Hu et al. [55]	AS	iPS cells	iPS cell establishment from a JAK2 mutated AS patient
Hu et al. [56]	AS	iPS cells	iPS cell establishment
Son et al. [57]	AS, SS, SLE	Hematopoietic cells, mesenchymal cells	Integrin-free iPS cell establishment
Son et al. [58]	BD	Hematopoietic precursor cells	Elevated expression of 8 genes, including CXCL1, in BD-iPS cell-derived hematopoietic precursor cells
Iizuka-Koga et al. [59]	SS	Dendritic cells	Functional DC differentiation from SS T cell clone-derived iPS cells
Wang et al. [60]	SSc	Fibroblasts	Normal collagen and intergrin expression in SSc iPS cell-derived fibroblasts
Gholami et al. [61]	SSc	Endothelial cells	Lower cadherin expression and defective tube formation in SSc iPS cell-derived ECs
Chen et al. [62]	SLE	iPS cells	iPS cell establishment from urinal tubular cells of SLE
Tang et al. [63]	SLE	iPS cells	Differentially expression between SLE and healthy donor iPS cells, including AK4 upregulation
De Angelis et al. [64]	SLE	iPS cells	iPS cell establishment from a patient with CNS lupus. Increased Erk, Akt, and oxidative stress -associated pathway in SLE iPS cells
Park et al. [65]	SLE	Cardiomyocytes	Decreased proliferation and increased levels of fibrosis and hypertrophy marker expression in SLE iPS cell-derived cardiomyocytes. Pathogenic effects of anti-Ro antibody on iPS cell-derived cardiomyocytes
Li et al. [66]	SLE	iPS cells	iPS cell establishment
Li et al. [67]	SLE	iPS cells	iPS cell establishment
Natsumoto et al. [20]	SLE	CD123^+^ dendritic cells	iPS cell establishment from SLE sisters. Enhanced IFN-alpha secretion in DCs due to *OASL* variant

References and abbreviations are the same as the main document

### Rheumatoid arthritis

Rheumatoid arthritis (RA) is characterized by polyarthritis arising from autoimmunity [68]. Notably, autoantibodies, such as anti-modified protein antibodies, emerge even before the clinical onset of the disease. Fibroblasts are also central players in the pathogenesis of RA. Synovial fibroblasts produce pro-inflammatory cytokines, such as IL-6 and contribute to bone destruction [68]. Several research groups successfully established iPS cells from fibroblast-like synoviocytes (FLSs) of patients with RA [48–50]. These iPS cells, sourced from FLSs, have been found comparable to those derived from the other cell types and have been shown to differentiate into functional cardiomyocytes [51]. The iPS cells derived from RA-FLSs stand as valuable tools for probing the pathogenic roles of FLSs in RA.

With respect to the application of RA patient-derived iPS cells, two reports have showcased diverse approaches. Kim et al. generated FLSs from three RA patients-derived iPS cells [52]. Their findings revealed that certain metabolites, such as nicotinamide (NAM), were more abundantly produced in the iPS cell-derived FLSs from patients with RA compared to those from patients with osteoarthritis (OA). As NAM promotes the proliferation of FLSs, it might be intricately linked to the pathogenesis of synovitis in RA. Another report focused on iPS cell-derived hepatocytes in 2D and 3D cultures, seeking to understand methotrexate-induced hepatotoxicity [53].

One challenge in this field is that obtaining FLSs from RA patients requires invasive procedures, such as needle biopsies or surgeries, presenting both ethical and technical issues. While iPS cell-derived FLSs might not mirror primary cells in all respects, there are numerous advantages to employing these in research. In this way, various cell lineages, beyond just immune cells, have been differentiated from RA patient-derived iPS cells, offering new avenues for the investigation of RA. In particular, iPS cell-derived FLSs hold promise for delving deeper into the pathogenesis of RA. Nonetheless, certain challenges persist, like ascertaining whether iPS cell-derived FLSs can consistently exhibit RA-specific phenotypes.

### Ankylosing spondylitis

Ankylosing spondylitis (AS) is another type of autoimmune arthritis, distinctively marked by sacroiliitis and spondylitis. The presence of *HLA-B27* is a significant genetic risk factor for AS, and it plays a central role in the pathogenesis of AS [69]. While only a few reports have described the generation of iPS cells from patients with AS [54–57], Layh-Schmitt et al. detailed the generation of iPS cells from the dermal fibroblasts of two patients with axial spondyloarthropathy (axSpA) [54]. Intriguingly, mesenchymal stem cells (MSCs) differentiated from axSpA patient-derived iPS cells exhibited elevated expression of several AS susceptibility genes implicated in bone formation, in contrast to healthy donor-derived iPS cells. Given that MSCs have the potential to differentiate into osteoblasts, axSpA patient-derived iPS cells present a valuable tool for exploring the connection between genetic risks and bone formation in the pathogenesis of AS.

### Behcet’s disease

Behcet’s disease (BD) is typified by mucocutaneous inflammation and uveitis. Son et al. successfully generated BD patient-derived iPS cells [58] and differentiated into hematopoietic precursor cells (HPSc) to serve as a model for BD. Notably, they demonstrated that HPSc from BD patient-derived iPS cells expressed eight BD-specific genes, including *CXCL1*, distinguishing them from those derived from both healthy and disease controls [58].

### Sjogren’s syndrome

Sjogren’s syndrome (SS) is characterized by autoimmune sialadenitis. Autoreactive T cells infiltrate the organs and trigger inflammation [70]. Iizuka et al. established iPS cells from SS patient-derived CD4^+^ T cell clones and subsequently differentiated these cells into DCs [59]. The researchers demonstrated that iPS cell-derived DCs promoted autoreactive M3R-specific CD4^+^T cell proliferation [59]. As a result, they suggested that these iPS cell-derived DCs might serve as antigen-presenting cells, paving the way for more focused research on antigen-specific T cell research.

### Systemic sclerosis

Systemic sclerosis (SSc) is marked by the skin and organ fibrosis, accompanied by autoimmunity and vasculopathy [71]. Beyond the roles of immune cells, fibroblasts and endothelial cells (ECs) are crucial to the pathogenesis of SSc, with genetic risk factors influencing these cells [71]. Wang et al. generated iPS cells from dermal fibroblasts of patients with SSc and compared collagen-related gene expression between iPS cell-derived fibroblasts and SSc dermal fibroblasts. Intriguingly, the iPS cell-derived fibroblasts exhibited normal expression levels of collagen and integrin genes, whereas the SSc dermal fibroblasts expressed higher levels of collagen-related genes [60]. The authors concluded that the reprogramming process involved in generating iPS cells normalized the epigenetic modification observed in SSc fibroblasts. Gholami et al. established two SSc patient-derived iPS cells and differentiated them into ECs [61]. Notably, these ECs from SSc patient-derived iPS cells displayed significantly reduced cadherin expression and demonstrated impairments in tube formation [61]. These results suggest the potential angiogenesis deficits in patients with SSc.

#### SLE

SLE is a quintessential autoimmune disease, affecting multiple organs. Manifestations often include mucocutaneous, musculoskeletal, renal, hematological, and neurological disorders [45]. The foundation of the pathogenesis of SLE is thought to be autoimmunity [45]. Immune complex deposition prompts the chemotaxis of immune cell, leading to inflammation and subsequent organ damage. Additionally, pro-inflammatory cytokines, especially type 1 IFNs, are instrumental in the pathogenesis of SLE [72]. Like other autoimmune diseases, SLE is usually polygenic. However, several reports on familial and child-onset SLE patients indicated the presence of patients with distinctive rare variants [72]. Instances of monogenic lupus have also been documented [73], and in certain patients, rare variants related to AGS can cause monogenic SLE [40]. Therefore, SLE patient-derived iPS cells are thought to harbor SLE-prone genetic backgrounds, making them valuable tools for studying the pathogenesis and distinct features of SLE.

Several research groups established SLE patient-derive iPS cells, and these cells have been used to analyze SLE-specific features in iPS cell-based studies [20, 57, 62–67]. Chen et al. established iPS cells from urinal renal tubular cells from four patients with SLE [62]. In a subsequent study, Tang et al. compared these SLE patient-derived with healthy donor-derived iPS cells using multi-omics analysis [63]. They identified numerous differentially expressed genes, miRNA, and proteins. Gene ontology analysis indicated that these differentially expressed genes and proteins were predominantly associated with mRNA processing and translation. Notably, they observed an upregulation of *AK4*, which is involved in nucleotide biosynthesis, in SLE patient-derived iPS cells [63]. The *AK4* gene is a target of miR-317a-5p, and this miRNA was found to be downregulated in SLE patient-derived iPS cells. The authors posited that this integrated analysis of miRNA, mRNA, and protein might not only offer insights into the pathogenesis of SLE, but could also lead to the discovery of novel diagnostic biomarkers for SLE. However, a potential limitation of this study is the lack of prior reports about the upregulation of AK4 or miR-317a-5p in primary cells from SLE patients. We believe that examining iPS cell-derived immune cells, rather than stem cells, could provide deeper insights into the pathogenesis of lupus.

De Angelis et al. generated iPS cells from dermal fibroblasts of a patient with central nervous system (CNS) lupus [64]. They employed mRNA profiling to compare these cells with CNS lupus patient-derived iPS cells from healthy donor-derived iPS cells and identified differentially expressed genes, which were involved in Erk and Akt signaling pathways. Additionally, they detected dysregulated miRNAs associated with oxidative stress [64]. This led them to conclude that SLE patient-derived iPS cells can serve as valuable tools for probing the molecular mechanisms underlying the disease.

Park et al. generated cardiomyocytes from SLE patient-derived iPS cells and observed structural and functional differences between these and cardiomyocytes derived from healthy donors [65]. Intriguingly, when exposed to serum from patients with active SLE, the ipS cell-derived cardiomyocytes exhibited increased rates of apoptosis, proliferation, and fibrosis. These effects were more pronounced in SLE patient-derived iPS cells. Moreover, adding an anti-Ro antibody exacerbated the expression of genes related to fibrosis, hypertrophy, and apoptosis. The authors suggested that these iPS cell-derived cardiomyocytes could employed as models for studying organ damage in SLE.

Recently, our group reported a study in which we established iPS cells from SLE patients with a familial history of the disease, specifically from sisters with SLE (referred to as SLE-sister-derived iPS cells) [20]. Using a previously described method, we differentiated these iPS cells into CD123^+^ DCs. We hypothesized that these DCs would serve as an effective model for both SLE and interferonopathy, given their machinery to produce type 1 IFNs, such as cytosolic receptors. We observed that CD123^+^ DCs, when differentiated from SLE sister-derived iPS cells, exhibited increased secretion of IFN-alpha upon exposure to dsRNA. Whole exome analysis of the SLE sister-derived iPS cells revealed rare variants in the *OASL* gene. Using genome editing techniques, we corrected this *OASL* 202Q variant to the wild-type 202R, leading to a reduction in IFN-alpha secretion. Conversely, when we introduced the 202Q mutation into healthy donor-derived iPS cells, there was an amplified IFN-alpha secretion in response to dsRNA. This highlighted the role of the *OASL* variant in augmenting RNA-related pro-inflammatory effects, thereby contributing to the pathogenesis of SLE. Our findings reinforce the idea that SLE patient-derived iPS cells can be instrumental in uncovering studies that offer genetic factors pivotal to the development of SLE. In this way, these SLE patient-derived iPS cell-based studies offer novel disease models for in-depth pathological studies.

## Conclusions

In conclusion, iPS cells can be generated from patients with various immune-related diseases. However, a standardized research strategy has yet to be established, particularly for the study of autoimmune diseases. Moreover, numerous challenges arise when trying to analyze immune-related diseases using iPS cell-based studies. Given that acquired immunity in autoimmune diseases is genetically influenced, it is essential to develop differentiation protocols for T and B cells to accurately study these diseases and the human immune system. To account for the individual variations in immune-related diseases, it would be beneficial to have an extensive stock of patient-derived iPS cell lines in cell banks. Nevertheless, numerous studies have highlighted disease-specific phenotypes of immune-related diseases using patient-derived iPS cells.

We also advocate for the benefits of iPS cell-based studies. First and foremost, iPS cells are instrumental in drug screening and discovery. Furthermore, cell-based therapies founded on iPS cells present a promising strategy. Immune regulatory cells, such as MSCs, can be differentiated from iPS cells, allowing for the production of several autologous MSCs. The emergence of chimeric antigen receptor (CAR)-T cells as a therapeutic strategy for autoimmune diseases, including SLE, has recently garnered attention [74]. Some studies even suggest the feasibility of deriving CAR T cells from iPS cells [75, 76]. iPS cells possessing a monoclonal T cell receptor gene can potentially be harnessed to produce iPS cell-derived CAR-T cells for treating immune-related diseases. Furthermore, the clinical application of iPS cells holds immense potential for the future. Given their regenerative properties, numerous clinical trials have utilized iPS cell-derived cells and tissues [77]. This regenerative capability could be invaluable in addressing irreversible organ damage in autoimmune disease patients, such as those with lupus nephritis. Additionally, HLA-homozygous iPS cells have been stored in the CiRA Foundation [77]. These cells could pave the way for antigen-specific therapies for autoimmune diseases in the coming years.

In this way, iPS cell-based research emerges as a novel and promising approach, both for understanding the origins of immune-related diseases and for developing potential clinical applications (Fig. 1).

## Data Availability

Not applicable.

## Abbreviations

iPS: Induced pluripotent stem
FD: Familial dysautonomia
IkB: I kappa B
IKBKAP: IkB kinase complex-associated protein
TCR: T cell receptor
MDM: Monocyte-derived macrophage
MCS-F: Macrophage colony-stimulating factor
MP: Macrophage
DC: Dendritic cell
GM-CSF: Granulocyte-macrophage colony-stimulating factor
IL: Interleukin
IFN: Interferon
FMF: Familial Mediterranean fever
CCL: Chemokine (C–C motif) ligands
CINCA: Chronic infantile neurological cutaneous and articular
NFkB: Nuclear factor-kappa B
OAS: Oligoadenylate synthetase
OPAID: OAS1-associated polymorphic autoinflammatory immunodeficiency
AGS: Aicardi-Goutieres syndrome
SAVI: STING-associated infantile vasculitis
ISG: Interferon stimulated gene
TORCH: Toxoplasmosis, Rubella, Cytomegalovirus, and Herpes simplex virus
NNS: Nakajo-Nishimura syndrome
ds: Double-stranded
SLE: Systemic lupus erythematosus
MDA: Melanoma differentiation-associated protein
OASL: 2′-5′-Oligoadenylate synthetase-like
cGAS: Cyclic GMP-AMP synthase
ISG: Interferon signature gene
ANC: Anti-neutrophil cytoplasmic antibody
PSMB: Proteasome (prosome macropain) subunit beta
RA: Rheumatoid arthritis
FLS: Fibroblast-like synoviocyte
NAM: Nicotinamide
OA: Osteoarthritis
AS: Ankylosing spondylitis
axSpA: Axial spondyloarthropathy
MSC: Mesenchymal stem cell
BD: Behcet’s disease
HPC: Hematopoietic precursor cell
SS: Sjogren’s syndrome
SSc: Systemic sclerosis
EC: Endothelial cell
CNS: Central nervous system
CAR: Chimeric antigen receptor
